# Optimization of Preparation Conditions for Lysozyme Nanoliposomes Using Response Surface Methodology and Evaluation of Their Stability

**DOI:** 10.3390/molecules21060741

**Published:** 2016-06-08

**Authors:** Zhipan Wu, Rongfa Guan, Fei Lyu, Mingqi Liu, Jianguo Gao, Guozou Cao

**Affiliations:** 1 National & Local United Engineering Lab of Quality Controlling Technology and Instrumentation for Marine Food, China Jiliang University, Hangzhou 310018, China; wuzhipanjl@126.com (Z.W.); mqliu524@163.com (M.L.); 2 Department of Food Science, Ocean College, Zhejiang University of Technology, Hangzhou 310014, China; lvfei_zju@163.com; 3 Inspection and Quarantine Center of Shandong Exit & Entry Inspection and Quarantine Burean, Qingdao 266002, China; china.gjg@163.com; 4 Ningbo Inspection and Quarantine Institute of Science and Technology, Ningbo 315000, China; caogz@nbciq.gov.cn

**Keywords:** lysozyme, nanoliposomes, response surface methodology, optimization, stability

## Abstract

The main purpose of this study was to optimize the preparation of lysozyme nanoliposomes using response surface methodology and measure their stability. The stabilities of lysozyme nanoliposomes in simulated gastrointestinal fluid (SGF), simulated intestinal fluid (SIF), as well as pH, temperature and sonication treatment time were evaluated. Reverse-phase evaporation method is an easy, speedy, and beneficial approach for nanoliposomes’ preparation and optimization. The optimal preparative conditions were as follows: phosphatidylcholine-to-cholesterol ratio of 3.86, lysozyme concentration of 1.96 mg/mL, magnetic stirring time of 40.61 min, and ultrasound time of 14.15 min. At the optimal point, encapsulation efficiency and particle size were found to be 75.36% ± 3.20% and 245.6 nm ± 5.2 nm, respectively. The lysozyme nanoliposomes demonstrated certain stability in SGF and SIF at a temperature of 37 °C for 4 h, and short sonication handling times were required to attain nano-scaled liposomes. Under conditions of high temperature, acidity and alkalinity, lysozyme nanoliposomes are unstable.

## 1. Introduction

Lysozyme (EC 3.2.1.17) is officially described as *N*-acetylhexosaminodase and is involved in every secretion, such as body fluids, and tissue of animal organisms [1]. It has also been isolated from some plants, bacteria, and bacteriophages [2]. This enzyme causes the cell-wall of susceptible bacteria to be hydrolyzed, such as Gram-positive bacteria, which increases their permeability and causes the cells to burst [3]. This lytic function of lysozyme on Gram-positive cell walls has been used in making pharmaceutical products for several years (to increase the natural defenses of the body, to treat pharyngalgia, to clean contact lenses, and in formulas for infant-feeding) in the pharmaceutical and food industry [4]. Many scientific papers have focused on the antibacterial properties of lysozyme and their use in food processing to better control bacterial pollution and fermentations in a variety of food products [2].

However, interaction of antimicrobial compounds with various food components reduces their efficacy against pathogens, and, thus, large antimicrobial concentrations are often required [5]. Methods that reduce interaction of antimicrobials with food components, e.g., encapsulation, have the capacity to increase antibacterial activity and stability in some food systems [5].

A significant effort towards these aims comprises the use of colloidal delivery systems such as liposomes and micro or nanoparticles [6,7]. Recently, liposomes—globose particles less than 1000 nm in size formed by lipid bilayers—have received much attention in research [8,9,10,11]. Liposomes can catch a large number of hydrosoluble substances and have been mainly used in pharmaceutical applications [5]. Recently, various food components and additives, including carotenoids and nisin Z, have successfully been encapsulated in liposomes [12,13] and used in food systems. The liposome enhances the stability of the encapsulated material by protecting them from the environment [11].

Response surface methodology (RSM) is a useful technique for analyzing interactions among various factors and exploring the relationships between the response and the independent variables [14]. It is a collection of statistical and mathematical techniques that has been used for developing, improving, and optimizing various processes [15,16]. As a powerful statistical tool, RSM has been successfully used in various fields of food chemistry such as in the optimization of anthocyanin hydrolysis from red wine and the optimization of the solvent extraction of phenolic compounds from beans and other plants [17,18,19].

The major objective of this research was to study the effect of the rate of phosphatidylcholine and cholesterol (*w*/*w*), lysozyme concentration (*w*/*v*), ultrasound and magnetic stirring time (min) on the encapsulation efficiency so as to determine the optimal conditions for making lysozyme nanoliposomes using RSM. Furthermore, nanoliposomes were examined in different environments for their evaluation of stability, especially in simulated gastric fluid (SGF) and simulated intestinal fluid (SIF).

## 2. Results and Discussion

### 2.1. Fitting the Model

The second order polynomial response surface model (Equation (2)) was suitable for the response variable (Yi). For the corresponding fitting of the models, the transformation of encapsulation efficiency was analyzed. The coefficients of polynomial equation were calculated by using this experimental data, which were used effectively for the prediction of encapsulation efficiency.

Table 1 displays the results from regression and variance analysis. The statistical significance of response surface models was assessed according to regression analysis and the analysis of variance (ANOVA). The estimated regression coefficients for the response variable, along with the adjusted R^2^ (adj-R^2^), corresponding R^2^, *p*-value, and lack of fit, are shown in Table 1.

From Table 1, it can be seen that the linear relationship between the dependent variable and independent variables is significant. The determination coefficient was used to evaluate the lack of fit of each model. The lack of fit showed that the models failed to represent the data in the experimental domain at the points which were not included in the regression [20]; lack of fit was also used to check the quality of the fitted models. The lack of fit of the EE was 0.28, which was not significant (*p* > 0.05), meaning that the data in the model were accurate.

An R^2^ value closer to unity indicated a better empirical model fit to actual data. The R^2^ value for the response variable of the EE was 0.97 which exceeded 0.80, demonstrating that the regression model was appropriate to explain the behavior, but the quality of the model cannot always be assured by a large R^2^ value. Regardless of whether the additional variable is statistically significant or not, increasing a variable in the model will always increase R^2^. Thus, it is better to use an adj-R^2^ to evaluate model adequacy [20]. R^2^ was 0.97 and Adj-R^2^ was 0.93, which prove that the model fits well with the experimental data and the model can be used to analyze and estimate the generated lysozyme nanoliposomes. In general, a CV exceeding 10% indicates that transformation in the mean value is high and is not suitable for developing a proper response model. The CV value for EE was found to be 4.97, which suggested the experiments had good reliability and repeatability.

### 2.2. Encapsulation Efficiency

The *p* values are used as a tool to check the significance of every coefficient [20]. The smaller the magnitude of *p* is, the more significant the corresponding coefficient. Values of *p* less than 0.05 indicate that model terms are significant [20].

**Figure 1:**
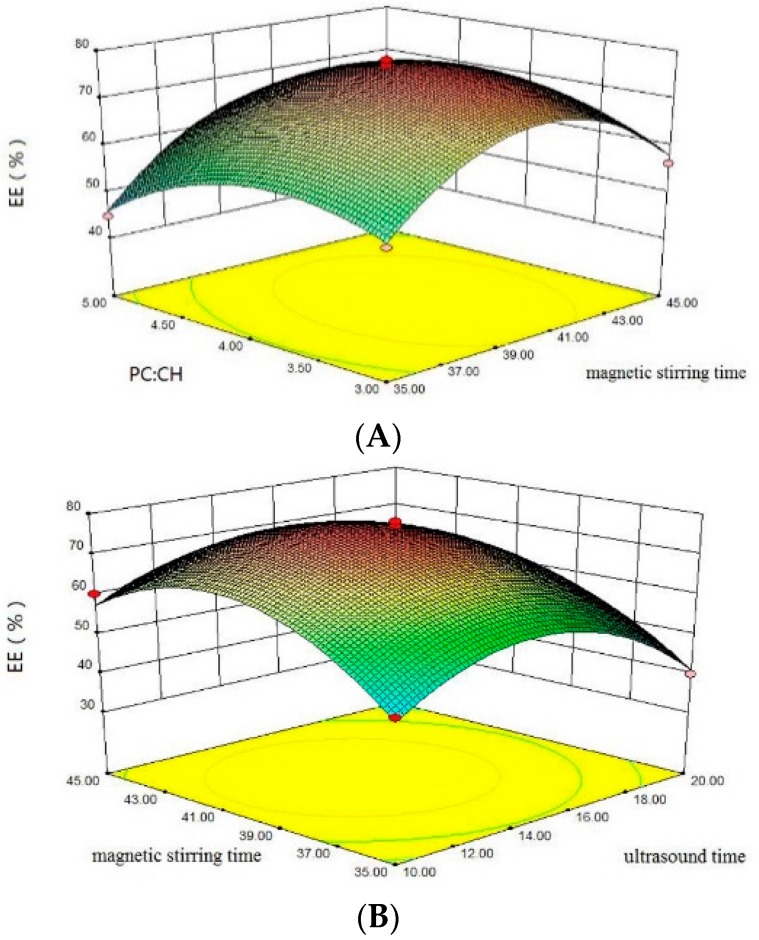
Response surface figures with the influence of the independent variables on encapsulation efficiency of lysozyme nanoliposomes. The effects of phosphatidylcholine-to-cholesterol ratio and magnetic stirring time are shown in (**A**) (ultrasound time = 15 min and lysozyme concentration = 2 mg/mL); the effects of magnetic stirring time and ultrasound time are shown in (**B**) (phosphatidylcholineto-cholesterol ratio = 4 and lysozyme concentration = 2 mg/mL).

The results in Table 1 show that the linear effects of phosphatidylcholine-to-cholesterol ratio, ultrasound time, and magnetic stirring time were significant (*p* < 0.05), while lysozyme concentration was not significant (*p* > 0.05). Influence of the independent variables on lysozyme nanoliposomes is demonstrated in Figure 1. The interactive effects of independent variables on the responses were further investigated by constructing three-dimensional response surface graphs and two-dimensional contour plots [21]. In accordance with Figure 1A, the encapsulation efficiency was increased with the increasing phosphatidylcholine-to-cholesterol ratio. It might be due to the fact that cholesterol can change the order of mobility of lecithin in the lipid bilayer, thus reinforcing the membrane stability [22]. For another, increasing the magnetic stirring time could increase the encapsulation efficiency (EE), allowing more lysozyme to be encapsulated in the nanoliposomes.

As shown in Figure 1B, the increase in ultrasound time led to an increase in the EE of lysozyme nanoliposomes. At longer ultrasound times, the EE was added because ultrasound causes the organic phase and water phase to form an emulsion, and more lysozyme is encapsulated in the vesicles. This can be shown that the higher phosphatidylcholine-to-cholesterol ratio, ultrasound time and magnetic stirring time, could increased the encapsulation efficiency.

### 2.3. Optimization

**Figure 2:**
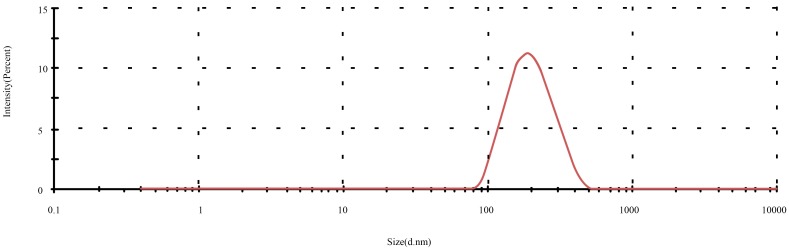
The particle size of the optimized lysozyme nanoliposomes.

**Figure 3:**
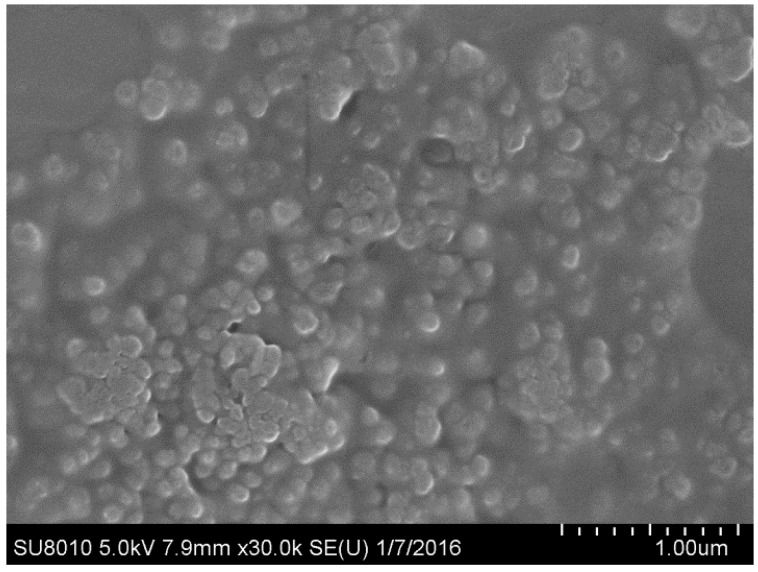
The morphology of the optimized lysozyme nanoliposomes.

After the effects of lysozyme concentration, PC/CH, ultrasound time, and magnetic stirring time on the preparation of lysozyme nanoliposomes were studied, the optimum ranges for each independent variable were determined in order to prepare lysozyme nanoliposomes with the highest EE. The optimum preparation conditions were as follows: phosphatidylcholineto-cholesterol ratio of 3.86, lysozyme concentration of 1.96 mg/mL, ultrasound time of 14.15 min, and magnetic stirring time of 40.61 min. The conditions provided the highest encapsulation efficiency (77%), and the experimental values had a good agreement with the predicted values, which showed that the optimized conditions of preparation were very reliable. Optimized lysozyme nanoliposomes were used for the measurement of particle size distribution (Figure 2). At this optimum point, particle size was found to be 245.6 nm ± 5.2 nm. The results indicated that the optimized liposomes were in the nanoscale. Scanning electron micrographs revealed that the produced lysozyme nanoliposomes had a regular spherical shape and formed and agglomerated in a fully spread manner (Figure 3). SEM analysis (Figure 3) showed that the particle size of the prepared nanoliposomes was approximately 250 nm.

### 2.4. Stability of Lysozyme Liposome

#### 2.4.1. Stability Test to pH

**Figure 4:**
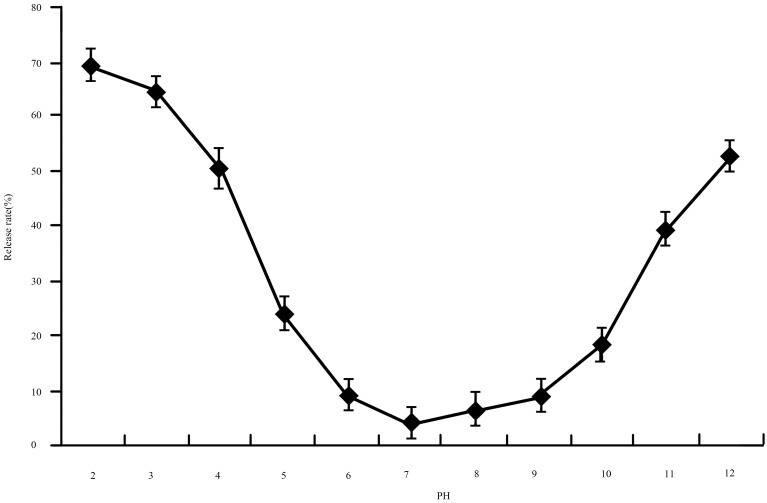
The effect of release ratio on lysozyme nanoliposomes under different pH.

pH does not stabilize liposomes, but contributes to stability or instability. Much research has indicated that the hydrolysis of phosphatidyl choline and phosphatidyl glycerol is affected by pH. At pH 1 or pH 12, phosphatidyl choline is hydrolyzed in a very short period of time. A series of pH values from 2.0 to 12.0, depending on practical requirements [23], were used for the study of stability. The release ratio in lysozyme nanoliposomes at different pH values is demonstrated in Figure 4. Results clearly showed that pH has a great impact on the release ratio of lysozyme nanoliposomes. At pH 2.0 and pH 12.0 on nanoliposomes, the release ratios were 69% and 53%, respectively, which were higher than the release ratio of pH 7.0. From Figure 4, it can be seen that there was a remarkable reduction in release ratio along with a drop in pH values until pH 7.0, while a neutral environment ensured the lysozyme nanoliposomes had good stability. This indicates that a pH value of 7.0 is beneficial for keeping lysozyme nanoliposomes.

#### 2.4.2. Thermostability Test

**Figure 5:**
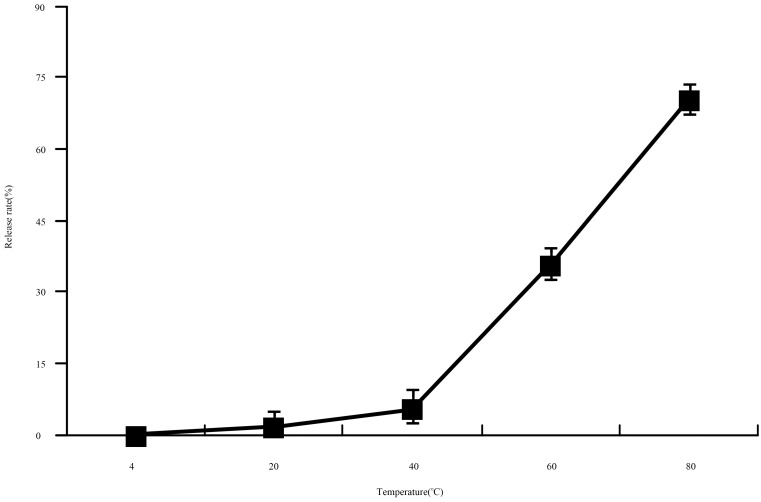
The effect of release ratio on lysozyme nanoliposomes at different temperatures.

Thermal treatment experiments were carried out at 4, 20, 40, 60 and 80 °C, all of which were used in industrial treatments [23]. The release rate of lysozyme nanoliposomes at different temperatures demonstrated that lysozyme nanoliposomes can resist thermal treatment under the temperature of 40 °C (Figure 5). The release rate clearly accelerates with an increase in temperature. Lysozyme nanoliposomes at 80 °C demonstrated the highest release rate, with more than 70% of lysozyme nanoliposomes ruptured within 30 min. Lysozyme nanoliposomes were relatively stable at 4–40 °C. The phase transition temperature of soya bean lecithin is 52 °C, and cholesterol may reduce the cell membrane fluidity when the cell membrane fluidity is excessive. It was suggested that lower temperature treatment could be an effective method to preserve lysozyme nanoliposomes.

#### 2.4.3. Effect of Sonication

**Figure 6:**
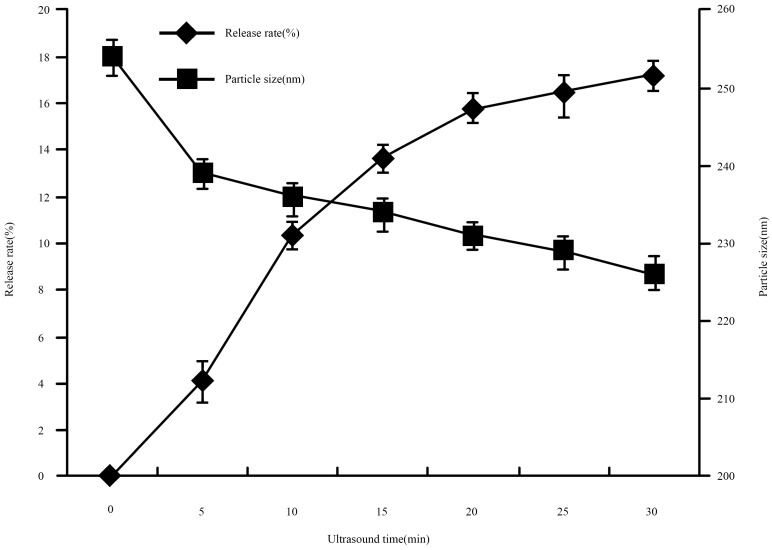
The effect of release ratio on lysozyme nanoliposomes at different ultrasound times.

Sonication is an effective method to develop a w/o emulsion in preparation, and to control and decrease the size of liposomes after the preparation of nanoliposomes. We evaluated whether applying ultrasound to the nanoliposomes resulted in the release of lysozyme from the nanoliposomes. The stability of lysozyme nanoliposomes was tested by an experiment examining the release ratio, as shown in Figure 6. After testing, we came to the conclusion that a significant increase in the release rate occurred when lysozyme nanoliposome was subjected to ultrasound, which showed that lysozyme outflowed from the nanoliposome after ultrasound. The release ratio was 10.31% after 10 min sonication on lysozyme nanoliposomes, which was higher than the release rate of 5 min of sonication. The effects of ultrasound suggested that nanoliposomes could present an interesting ultrasound-sensitive induced release method and could be a promising candidate for ultrasound-sensitive drug delivery systems [24]. After 5 min of ultrasound on samples, the particle size of lysozyme nanoliposomes changed significantly, and the release ratio on lysozyme nanoliposomes was low. Therefore, the 5 min sonication time on lysozyme nanoliposomes may be suitable for decreasing the size of liposomes based on high EE.

#### 2.4.4. *In Vitro* Release of Lysozyme from Nanoliposomes

*In vitro* release researches are often conducted to evaluate how a drug delivery system might work in ideal conditions which might provide some indication of its *in vivo* activity.

**Figure 7:**
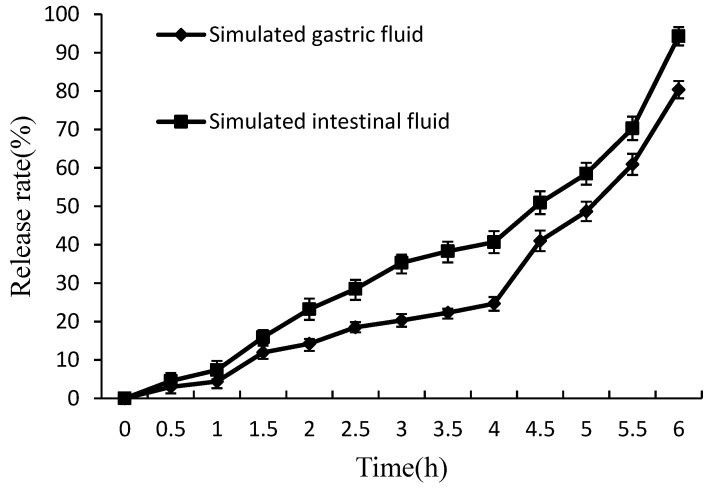
The effect of SGF and SIF on nanoliposomes.

Due to the probable degradation of encapsulated bioactive components by high acid and enzymes in SGF, the protective effect of the lipid bilayer on the core materials is of much importance [25]. Figure 7 shows that about 25% of the lysozyme was outflowed from nanoliposomes at 4 h in SGF. The lipid bilayer is excellent at keeping the encapsulated lysozyme. The lysozyme nanoliposomes demonstrated an acceptable stability and may be suitable for use in antibacterial agent. The susceptibilities of liposomes in SIF present significant effects of pancreatic lipase [25]. Figure 7 shows that lysozyme reached the release rates of 40% after 4 h in SIF, and the release of lysozyme was a slow and continuous process during the study period. A slow and sustained release is preferred in SIF, since more carrier lipids can be digested and a greater fraction of the bioactive components can be solubilized by micelles and adsorbed by enterocytes [26].

## 3. Material and Methods

### 3.1. Materials

Lysozyme (from chicken egg white) was purchased from Shanghai Jingchun Biochemical Technology Company (Shanghai, China). Cholesterol (CH) and phosphatidylcholine (PC) were obtained from Beijing Shuangxuan Microorganism Co. Ltd. (Beijing, China). The type of PC is soya bean lecithin. PC lipid is electrically neutral, so electrostatic interaction is difficult to achieve between lysozyme and PC hydrophilic groups. On the other hand, the hydrophobic interaction between lysozyme and PC lipid membrane is also unlikely, because four disulfide bonds within the lysozyme molecule prevent the exposure of hydrophobic amino acid residues. Therefore, PC membrane is stable for lysozyme encapsulation. Pepsin and steapsin were purchased from Shanghai Jingchun Biochemical Technology Co. (Shanghai, China). Chloroform and diethyl ether were received from Shanghai Changcheng Instrument & Reagent Company (Shanghai, China). All other chemicals were of analytically pure grade. The water used for all experiments was of national standard.

### 3.2. Methods

#### 3.2.1. Preparation of Lysozyme Nanoliposomes

Lysozyme nanoliposomes were prepared by using a reverse phase evaporation method. This method can encapsulate large macromolecules, with high encapsulation efficiency [27]. Through this method, the lysozyme liposomes are formed by water-in-oil emulsions with a certain amount of phospholipids, cholesterol and buffer, and lysozyme is dissolved in the phosphate buffer (PBS, 0.20 M, pH 6.8) [28,29]. The water-in-oil emulsions were pretreated by magnetic stirring and ultrasound, and these parameters would be optimized by RSM. The PC and CH are firstly dissolved in chloroform-diethyl ether to form a layer of thin film, secondly the solvents are removed by a rotary evaporator under reduced pressure at 37 °C. The thin film is re-suspended in 30 mL phosphate buffer, and continues to evaporate for about 20 min.

#### 3.2.2. Encapsulation Efficiency Determination

The encapsulation efficiency (EE) of lysozyme nanoliposomes is primarily aimed at determining the concentration of unencapsulated lysozyme in nanoliposome and encapsulated lysozyme in the system. The lysozyme nanoliposomes are separated from the system by a freeze centrifuge. Then, 0.5 mL nanoliposomes solution is taken and centrifuged at 15,000× *g* for 30 min at 4 °C. The same nanoliposomes solution is ruptured using a sufficient volume of film breaking reagent, and the total amount of lysozyme is determined by an ultraviolet spectrophotometer.

The activity of the encapsulated and free lysozyme was measured to estimate the EE (%) according to Equation (1) [30]:

(1) $$\mathrm{EE}\%=\frac{P_{2}-P_{1}}{P_{2}}\times 100$$

where P_1_ is the quantity of free lysozyme, and P_2_ is the total quantity of lysozyme present in 0.5 mL of lysozyme nanoliposomes.

#### 3.2.3. Particle Size

The particle size was measured by a particle size analyzer (Mastersizer 2000 instrument, Malvern, UK). Test was carried out in the range between 0.01 μm and 1000 μm, under the following requirements: refractive index of water is 1.33 and routine calculation model for irregular particles. The obtained data were averaged by software (Mastersizer 2000, ver. 5.20 from Malvern Company). The determination was measured three times for every sample.

#### 3.2.4. Experimental Design and Optimization

When desired responses are impacted by many interactions and other factors, response surface methodology (RSM) is an efficient tool for optimizing the preparation. Four factors with three levels applying face-centered central composite design were managed using Design-Expert 8.0.6 Software (Stat-Ease, Inc., Minneapolis, MN, USA) with the next treatment factor combinations to ascertain the optimum indexes for lysozyme nanoliposomes as shown in Table 2. The optimization design provided a total of 29 experimental groups. The range of level values of each factors was determined based on the single-factor experiments parameters [31].

By using a second order polynomial model, the response could be linked with the selected variables. In this research, a second order polynomial (Equation (2)) was used to form response surfaces.

(2) $${\overset{\wedge }{Y}}_{i}=b_{0}+\sum_{i}b_{i}x_{i}+\sum_{i}b_{\mathrm{ii}}x_{i}^{2}+\sum_{i\neq j}b_{ij}x_{i}x_{j}$$

where Ŷ_i_ represents the predicted responses, X_i_ and X_j_ are the coded values of independent variables, b_0_ is the intercept coefficient, b_i_ are the linear coefficients, b_ii_ are the squared coefficients, and b_ij_ are the interaction coefficients [32]. Statistical significance of the data in the regression equations was measured. The significant data in the model were determined by analysis of variance (ANOVA) for each response. The quality of the model was checked based on R^2^ and adjusted R^2^. The desired objectives for each variable and response were selected. All the independent variables were maintained within the range while the responses were either minimized or maximized.

#### 3.2.5. Stability of Lysozyme liposome

##### Stability Test to pH

The phosphate buffer solutions with hydrochloric acid and sodium hydroxide were adjusted to pH 2.0, 3.0, 4.0, 5.0, 6.0, 6.8, 7.0, 8.0, 9.0, 10.0, 11.0, and 12.0 to test the effect of pH on the stability of lysozyme nanoliposomes. Then, 3 mL of buffer solution at different pH was dissolved in standard lysozyme nanoliposomes (3 mL). After 30 min, encapsulation efficiency of each sample was experimented. The release ratios were obtained via Equation (3).

(3) $$\mathrm{Release}\;\mathrm{ratio}\%=\left(1-\frac{{\mathrm{EE}}_{i}}{{\mathrm{EE}}_{6.8}}\right)\times 100$$

where EE_6.8_ is the encapsulation efficiency of lysozyme nanoliposomes after reactivity for the standard solution (pH 6.8) and EE_i_ is the encapsulation efficiency of lysozyme nanoliposomes after reactivity for the solution of different pH values.

##### Thermostability Assay

The thermal stability of lysozyme nanoliposomes was evaluated at five different temperatures (4, 20, 40, 60, and 80 °C) at the same pH value (pH 6.8). After 30 min, lysozyme nanoliposomes were taken out and the encapsulation efficiency of each sample was measured. The release ratios were obtained via Equation (4).

(4) $$\mathrm{Release}\;\mathrm{ratio}\%=\left(1-\frac{EE_{T}}{EE_{4}}\right)\times 100$$

where EE_4_ is the encapsulation efficiency of lysozyme nanoliposomes after reactivity for standard temperatures (4 °C) and EE_t_ is the encapsulation efficiency of lysozyme nanoliposomes after reactivity for different temperatures.

##### Effect of Sonication

Lysozyme nanoliposomes (30 mL) were placed in a 50 mL beaker and were sonicated by a probe sonicator (VCX400, Sonics & Material, Inc., Newtown, CT, USA) in an ice bath. Samples of 3 mL were taken out at every five minutes. Encapsulation efficiency of withdrawn samples was measured. The release ratios were calculated.

##### *In Vitro* Release of Lysozyme from Nanoliposomes

SGF and SIF were prepared according to a previous study [33]. SGF was composed of hydrochloric acid (0.10 M), pepsin and deionized water. The pH value of SGF reached 1.3 via hydrochloric acid (0.10 M). SIF was composed of sodium hydroxide (0.10 M), potassium dihydrogen phosphate (6.8 mg/mL), deionized water and trypsin (10 mg/mL). The pH reached 7.5 by using sodium hydroxide (0.10 M). A certain amount of lysozyme liposomal samples (15 mL) was mixed with the equal volume of SGF or SIF in a 100 mL shaker, and then placed on in a shaking table which is 150 rpm and 37 °C. Every half hour until 6 h, an aliquot of the mixture was taken out of the 100 mL shaker, and placed in a 10 mL tube. The release of lysozyme from nanoliposomes was indicated by release ratio. Each experiment was carried out in three parallel experiments, and average values were obtained. The release rate was obtained via Equation (5).

(5) $$\mathrm{Release}\;\mathrm{ratio}\%=\left(1-\frac{EE_{t}}{EE_{0}}\right)\times 100$$

where EE_0_ is the encapsulation efficiency of lysozyme nanoliposomes before reactivity and EE_t_ is the encapsulation efficiency of lysozyme nanoliposomes after reactivity.

#### 3.2.6. Statistical Analysis

The results were written as mean ± standard deviation on the basic of three independent experiments. The statistical study was carried out with SPSS (International Business Machines Corporation, Armonk, NY, USA, version 17.0 for windows).

## 4. Conclusions

The effects of the phosphatidylcholine-to-cholesterol ratio, concentration of lysozyme, magnetic stirring time and ultrasound time on preparing lysozyme nanoliposomes were studied. Second order polynomial models were established to estimate encapsulation efficiency. Examining the phosphatidylcholine-to-cholesterol ratio, ultrasound time, and magnetic stirring time helped to determine the encapsulation efficiency. Optimization of parameters determined the optimum formation conditions, which were as follows: a lysozyme concentration of 1.96 mg/mL, a phosphatidylcholine to cholesterol ratio of 3.86, magnetic stirring time of 40.61 min, and ultrasound time of 14.15 min. With these criteria, the experimental size and encapsulation efficiency of the lysozyme nanoliposomes were 245.6 nm ± 5.2 nm and 75.36% ± 3.20%, which approached the predicted value. The stabilities of lysozyme nanoliposomes in pH, temperature and sonication treatment time were tested. The lysozyme nanoliposome showed an acceptable stability. Furthermore, nanoliposomes were measured in SGF and SIF for their stability. The lysozyme nanoliposomes demonstrated certain stability in SGF and SIF at a temperature of 37 °C for 4 h. The results suggest the prepared and optimized lysozyme nanoliposomes were stable and may be suitable for use in future research.

## Figures and Tables

**Table 1:** ANOVA and regression coefficients of the second-order polynomial model for the response variable (actual value).

Source	DF	EE%		
		Coefficient	Sum of Squares	*p* Value
Model	14	76.56	3098.91	<0.0001
Linear				
X_1_	1	−2.58	80.08	0.0069
X_2_	1	−0.34	1.40	0.6823
X_3_	1	−4.73	268.85	<0.0001
X_4_	1	3.46	143.52	0.0008
Quadratic				
X_1_^2^		−8.38	455.51	<0.0001
X_2_^2^		−9.84	628.38	<0.0001
X_3_^2^		−14.63	1388.35	<0.0001
X_4_^2^		−14.47	1357.68	<0.0001
Interaction				
X_1_X_2_		−0.55	1.21	0.7035
X_1_X_3_		−0.25	0.25	0.8624
X_1_X_4_		1.75	12.25	0.2367
X_2_X_3_		1.73	11.90	0.2432
X_2_X_4_		−1.35	7.29	0.3565
X_3_X_4_		−1.63	10.56	0.2703
Residual	14		112.24	
Lack of fit	10		92.76	0.2797
Pure error	4		19.47	
Total	28		3211.15	
R^2^		0.9650		
Adj-R^2^		0.9301		
CV		4.97		

**Table 2:** Independent variables and their levels in the experimental design.

Independent Variables	Symbols	Code Levels		
		−1	0	1
PC/CH (*w*/*w*)	X_1_	3	4	5
Lysozyme concentration (*w*/*v*)	X_2_	1	2	3
Ultrasound time (min)	X_3_	10	15	20
Magnetic stirring time (min)	X_4_	35	40	45

## Abbreviations

The following abbreviations are used in this manuscript:

SGF: Simulated gastrointestinal fluid
SIF: Simulated intestinal fluid
RSM: Response surface methodology
PC: Phosphatidylcholine
CH: Cholesterol
EE: Encapsulation efficiency
ANOVA: Analysis of variance
SEM: Scanning electron microscope
